# Integrated hydrothermal and microbial valorization of almond shells into xylooligosaccharides and cellobionic acid

**DOI:** 10.1186/s40643-026-01125-1

**Published:** 2026-09-26

**Authors:** Sumit Sharma, Hamidreza Shapouri, Shirin Kazemzadeh Pournaki, Daniela Barile, Takao Kasuga, Zhiliang Fan

**Affiliations:** 1 Department of Biological and Agricultural Engineering, University of California, Davis, One Shields Avenue, Davis, CA 95616 USA; 2 Department of Food Science & Technology, University of California, Davis, One Shields Avenue, Davis, CA 95616 USA; 3 Department of Plant Pathology, University of California, Davis, One Shields Avenue, Davis, CA 95616 USA

**Keywords:** Almond shell, Hydrothermal pretreatment, Xylooligosaccharides, *Thermothelomyces heterothallica*, Cellobionic acid

## Abstract

Almond shells are abundant lignocellulosic by-products with great potential for sustainable valorization. This study developed an integrated biorefinery process to produce xylooligosaccharides (XOS) and cellobionic acid (CBA) through hydrothermal pretreatment and fungal simultaneous saccharification and fermentation. Pretreatment under subcritical water conditions (165.9–194.1 ℃, 5.9–34.1 min) was optimized using response surface methodology and single-variable analysis. The optimal condition, selected to maximize XOS while minimizing xylose formation, was 180 ℃ for 8 min with 10% (w/v) solids, producing 18.1 ± 0.1 g/L XOS with a yield of 68.9 ± 0.3%. Low xylose (0.4 ± 0.0 g/L) and negligible glucose formation indicated selective oligomer production while preserving cellulose. The hydrolysate contained mainly short-chain XOS, with degrees of polymerization ranging from DP2 to DP10, and was particularly enriched in xylotriose and xylotetraose. Pretreated solids containing cellulose (15 g/L, ~ 46.3 mM) were converted to CBA using the engineered thermophilic fungus *Thermothelomyces heterothallica* TH-11 at 40 ℃ without enzyme addition. CBA production reached 31.8 ± 0.6 mM with a yield of 89.0 ± 0.5% based on cellulose consumed. These results demonstrate an environmentally friendly strategy for converting almond shells into value-added oligosaccharides and sugar acids, supporting circular bioeconomy development in the agri-food sector.

**Supplementary Information:**

The online version contains supplementary material available at https://doi.org/10.1186/s40643-026-01125-1.

## Introduction

Almond shells constitute the major lignocellulosic by-product of the almond processing industry. Despite their abundance, almond shells are currently used mainly for low-value applications such as combustion, charcoal production, and as reinforcing materials (Boulika et al. 2022; Demir 2025; El Mashad et al. 2022; Meadows 2023). However, their composition, containing approximately 26–38% cellulose, 23–28% hemicellulose, and 21–29% lignin (Gil-Guillén et al. 2024; Li et al. 2018),makes them an attractive feedstock for producing value-added bioproducts. Developing efficient conversion strategies for almond shells could therefore improve both resource utilization and economic sustainability in the almond industry.

Due to their woody structure, almond shells are highly recalcitrant to bioconversion and typically require intensive pretreatment. Kacem et al. (2016) employed a two-stage pretreatment using 4% NaOH followed by 1% H₂SO₄ and achieved approximately 6 g/L ethanol after enzymatic hydrolysis and fermentation with *Saccharomyces cerevisia*e (Kacem et al. 2016). Gong et al. (2011) identified an optimal sequential pretreatment involving hydrothermal treatment at 195 ℃ for 30 min, followed by 50% ethanol-water pretreatment at 195 ℃ for 20 min to maximize fermentable sugar production (Gong et al. 2011). Morales et al. (2020) studied the hydrothermal pretreatment for xylooligosaccharides production. They combined hydrothermal pretreatment with either organosolv or alkaline delignification before saccharification at a relatively high enzyme loading of 20 FPU/g solids (Morales et al. 2020). Across these studies, effective pretreatment generally required two pretreatment steps, increasing process complexity, and cellulase needed for enzymatic saccharification remained a significant cost driver. Therefore, there is a clear need for an integrated and sustainable biorefinery approach that can simultaneously valorize multiple biomass components under mild conditions while minimizing external inputs. We hypothesized that hydrothermal pretreatment of almond shells could selectively recover hemicellulose-derived XOS while preserving cellulose in the solid fraction, and that the resulting cellulose-rich solids could subsequently be converted to CBA by the engineered cellulolytic fungus *Thermothelomyces heterothallica* without exogenous enzyme addition. In this context, the present study aims to develop a simplified, enzyme-free process that couples hydrothermal pretreatment with fungal bioconversion to efficiently produce both XOS and cellobionic acid from almond shells, thereby enhancing overall resource utilization and economic viability.

XOS are high-value functional prebiotics that stimulate beneficial intestinal microorganisms and exhibit additional biological activities, including antioxidant, anti-inflammatory, antimicrobial, and antitumor effects (Huang et al. 2022; Samanta et al. 2015; Valladares-Diestra et al. 2023). They can be produced from the xylan fraction of the lignocellulosic biomass. Various pretreatment methods, including dilute acid, alkali, ammonia, organic solvents, and supercritical CO₂, have been investigated for almond shells (Brleković et al. 2024; Chamorro et al. 2023; Dávila et al. 2021; Gong et al. 2011; Li et al. 2025; Nitsos et al. 2018; Singh et al. 2019b). Among them, hydrothermal pretreatment is particularly attractive because it uses only water and avoids introducing corrosive chemicals into downstream processing, typically conducted at 160–200 ℃ for 5–30 min (Ilanidis et al. 2021; Jimenez-Gutierrez et al. 2021; Lee et al. 2023; Singh et al. 2019a; Zerback et al. 2022; Zhu et al. 2023). Hydrothermal pretreatment promotes hemicellulose solubilization through the autoionization of water while largely preserving cellulose and lignin (Scapini et al. 2021). However, conditions optimized for XOS production are relatively mild and leave most cellulose and lignin intact. As a result, the remaining solids are underutilized, and reports on further valorization beyond glucose production are limited (Morales et al. 2020).

Cellobionic acid (CBA), the aldonic acid derivative of cellobiose, has broad applications in the food, pharmaceutical, and chemical industries (Bieringer et al. 2023). It can be produced via either chemical or biochemical routes using cellobiose as a starting substrate (Bieringer et al. 2024; Chávez Morejón et al. 2023; Liu et al. 2024; Oh et al. 2022). However, the high cost of cellobiose limits CBA’s competitiveness. Yoo et al. (2022) tested wastepaper as a cheaper feedstock, achieving 23 g/L cellobiose via cellulase hydrolysis and 24 g/L CBA using recombinant *Pseudomonas taetrolens* (Yoo et al. 2022). Nevertheless, cellulase dependency and relatively low overall yields remain major limitations.

To address these challenges, cellulolytic fungi have been engineered to directly convert cellulosic biomass into CBA without exogenous enzyme addition. *Neurospora crassa* 2489, which naturally produces a full suite of cellulases, was engineered for CBA production. *Neurospora crassa* HL10, engineered by disrupting genes associated with cellobiose and cellobionate utilization and overexpressing a laccase gene from *Botrytis aclada*, produced CBA from pretreated wheat straw with yields approaching 90%. Similarly, *Thermothelomyces heterothallica* TH-11, a genetically engineered industrial cellulase producer, was developed through deletion of β-glucosidase, phosphorylase, and transporter genes involved in CBA metabolism (unpublished data). In the present study, TH-11 was applied directly to hydrothermally pretreated almond shells to produce CBA without additional pretreatment or exogenous cellulase. This work aims to establish an integrated and enzyme-free biorefinery process that combines XOS production with fungal CBA bioconversion, thereby improving overall biomass valorization and process sustainability.

## Materials and methods

### Raw material, chemicals, and microorganisms

Almond shells were collected from the Mariani Orchards, California, during the harvesting season in the year 2023 and stored at −20 ℃ until use. All chemicals used in this study were of analytical grade. Ethanol, glucose, sodium acetate, sodium formate, ammonium sulfate, potassium chloride, potassium phosphate, and magnesium sulfate were sourced from Fisher Scientific (Pittsburgh, Pennsylvania, USA). Sulfuric acid (72% w/w) was obtained from Alfa Aesar (Ward Hill, Massachusetts, USA). Xylose, furfural, 5-hydroxymethylfurfural (5-HMF), Avicel PH-101, and calcium carbonate were purchased from Sigma-Aldrich (Missouri, USA). Xylooligosaccharide standards, including xylobiose (DP2, > 95%), xylotriose (DP3, > 90%), xylotetraose (DP4, > 95%), xylopentaose (DP5, > 95%), and xylohexaose (DP6, > 95%), were obtained from Megazyme (Bray, County Wicklow, Ireland). Charcoal (Norit SA3) was purchased from Fisher Scientific (Rochester, New York, USA). Cellobiose was procured from Biosynth (Louisville, Kentucky, USA), while cellobionic acid was obtained from Synthose (Concord, Ontario, Canada). Commercial enzymes, including cellulase (≥ 700 U/g) from *Trichoderma reesei* and β-glucosidase (≥ 750 U/g) from *Aspergillus niger*, were supplied by Sigma-Aldrich (Missouri, USA). The engineered *T. heterothallica* TH-11 was constructed in the lab.

## Extractive-free almond shells preparation

Almond shells were air-dried at room temperature for 16–24 h, ground into powder, and sieved to obtain the + 25/−140 mesh fraction for experiments. Biomass composition was analyzed using the two-step hydrolysis NREL protocol (Sluiter et al. 2011). Extractive-free biomass was prepared following NREL/TP-510-42619 and previous studies (Kasangana et al. 2020; Sluiter et al. 2008; Tajmirriahi et al. 2021). For aqueous extraction, 10 ± 0.5 g of air-dried almond shells were mixed with 200 mL of distilled water in a 500 mL sealed bottle and incubated in a 50 °C water bath for 4 h with gentle agitation. The slurry was shaken every 30 min, then homogenized, filtered, and rinsed with 100–200 mL of distilled water. The recovered solids were air-dried in a fume hood for 24–48 h. The water-extracted biomass was further treated with 100 mL of 95% (v/v) ethanol under the same conditions for 4 h to remove remaining extractives. After extraction, the solids were washed with 100 mL of distilled water and air-dried for 24–48 h. The resulting extractive-free biomass was used for hydrothermal pretreatment.

## Optimization of the operating conditions for hydrothermal pretreatment

Approximately 0.7 ± 0.1 g of extractive-free biomass and 6.3 ± 0.1 g of distilled water were loaded into 14 mL stainless steel tube reactors. Reactors, prepared in triplicate, were sealed and soaked overnight. Samples were then pretreated at the specified temperature and residence time. To rapidly reach the target temperature, reactors were first preheated in a 210 °C oil bath, then transferred to a second oil bath maintained at the desired temperature. After pretreatment, reactors were cooled in an ice bath (0–4 °C) to stop the reaction. The liquid fraction was separated by filtration and used for sugar and XOS analyses.

Pretreatment conditions were optimized using response surface methodology to evaluate the interaction between temperature and time. A two-factor design was applied with temperature ranging from 170 to 190 °C and reaction time from 10 to 30 min. After determining the optimal conditions, the XOS-to-xylose ratio was further optimized by varying reaction time (4, 8, 12, 16, and 20 min) to identify the duration that maximized the ratio.

## Pretreatment in Parr reactors

Following optimization in the tube reactor, hydrothermal pretreatment was conducted in a 200 mL Parr reactor and a 1 L Parr 4843 reactor (both Parr Instruments, USA) to compare pretreatment efficiency across various reactor sizes and to generate sufficient pretreated solids for subsequent fermentation. In the 200 mL system, ~ 10 ± 0.5 g biomass was mixed with 90 ± 0.5 g distilled water. After pre-soaking, pretreatment was conducted at 180 °C for 8 min. Heating included a ramp from 21 to 100 °C over 7 min, then from 100 to 180 °C over 10 min.

In the 1 L reactor, 50 ± 2.0 g of almond shell biomass was combined with water to obtain 10% (w/w) solids. The temperature ramp was slower, increasing from 20 to 100 °C over 14 min and then to 180 °C over 28 min, with agitation at 100 rpm to ensure mixing.

In both systems, 180 °C was maintained for 8 min. After pretreatment, the reactor was rapidly cooled in an ice bath to quench reactions. The liquid fraction was collected for XOS and xylose analysis, while the solid fraction was washed, air-dried, and used for CBA production.

## Enzymatic hydrolysis of raw and pretreated almond shells

To evaluate the impact of pretreatment on biomass recalcitrance and cellulose digestibility, both the raw (extractive-free) and pretreated almond shells were subjected to enzymatic hydrolysis, with microcrystalline cellulose (Avicel) included as a reference substrate. Enzymatic hydrolysis was carried out using a commercial cellulase preparation supplemented with β-glucosidase under two enzyme loadings: (i) 15 FPU cellulase combined with 60 U β-glucosidase/g cellulose, and (ii) 60 FPU cellulase combined with 60 U β-glucosidase/g cellulose. For each experiment, 0.375 g of cellulose-equivalent solids (corresponding to 15 g/L or 92.6 mM cellulose or glucose equivalent) from raw biomass, pretreated biomass, or Avicel was suspended in 25 mL of 50 mM citrate buffer (pH 4.8) in serum bottles. Sodium azide (0.1%, w/v) was added to inhibit microbial growth. The sealed serum bottles were incubated at 50 °C in a rotary shaker rotating at 180 rpm for 7 days. Samples were collected at 24 h intervals; the released sugars were quantified by HPLC to determine cellulose hydrolysis yields.

### CBA production from pretreated almond shells

The fungal seed culture was prepared by inoculating 1 mL of *T. heterothallica* TH-11 stock culture into 50 mL of sterile rich medium containing yeast extract (6 g/L), (NH_4_)_2_SO_4_ (4.62 g/L), KCl (0.52 g/L), KH_2_PO_4_ (7.48 g/L), glucose (10 g/L), MgSO_4_ (2 mM), and trace elements, followed by incubation at 37 °C and 230 rpm. Seed cultures grown for 16–20 h were used as inoculum. Fermentation was initiated by transferring the 16–20 h seed culture into 50 mL flasks containing Vogel’s medium supplemented with pretreated solids (46.3 mM, equivalent to 15 g/L cellulose) and xylose (10 g/L), with a final inoculum size of 4% (v/v). Cultures were incubated at 40 °C and 230 rpm for 7 days. Samples were collected at various intervals for CBA and cellobiose analysis.

## Analytical method

For XOS analysis, a fraction of the hydrolysate was acidified to 4% H_2_SO_4_ and autoclaved at 120 ℃ for 60 min to hydrolyze XOS into xylose. The acidic hydrolysate was neutralized and analyzed for xylose using HPLC with a CarboSep CHO-87P column (Concise Separations, USA) operated at 80 °C with water (0.5 mL/min) for 30 min. The XOS concentrations were calculated by subtracting the monomeric xylose concentration from the post-hydrolysis xylose concentration.

Fermentation samples were boiled for 5 min and centrifuged at 13,000 rpm for 5 min. The supernatant was neutralized by adding Tris-HCl (pH 8.0) in a 4:1 (sample: buffer) ratio. Hydrolysate was acidified by adding 10% H_2_SO_4_ at a ratio of 10:1 (sample: acid). Following filtration, neutralized samples were analyzed for cellobiose and cellobionate concentrations using a CarboSep CHO-87 C column (Concise Separations, USA) operated at 80 °C with a 3 mM CaCl_2_ (0.6 mL/min) for 25 min. Acidified samples were analyzed for furfural, 5-HMF, formic acid, and acetic acid concentrations using an IC Sep Ion-300 column (Concise Separations, USA), operated at 60 °C using a 5 mM H_2_SO_4_ (0.5 mL/min) as the mobile phase, run for 60 min.

Filter paper activity (FPU/mL) was determined according to the NREL laboratory analytical procedure (NREL/TP-510-42628) (Adney and Baker 1996). Briefly, enzyme samples were incubated with 50 mg of Whatman No. 1 filter paper in 0.05 M citrate buffer (pH 4.8) at 50 °C for 60 min. Reducing sugars released during hydrolysis were quantified using the dinitrosalicylic acid (DNS) method, and cellulase activity was calculated based on a glucose standard curve.

To determine the degree of polymerization of XOS, the hydrolysate was prepared in 3 batches described further: (1) Tube reactor hydrolysate, (2) Parr reactor hydrolysate, and (3) Purified Parr reactor hydrolysate. XOS was purified using the charcoal-assisted adsorption and ethanol-assisted desorption method (Chinbat et al. 2024; Ratnadewi et al. 2025; Sonkar et al. 2023; Zhang et al. 2018) optimized for XOS recovery (data not shown). In brief, a 200 mL crude hydrolysate was mixed with 6% charcoal and transferred to the column (500 mL) containing a cotton plug at the bottm. The clean hydrolysate was collected, and then the adsorbed oligosaccharides were eluted by passing through 200 mL of 20% ethanol (3 times). All the purified liquids were concentrated by freeze-drying at -50 ℃ under a vacuum at 0 bar pressure for 72 h. After freezing, a purified powder was formed.

XOS chain-length distribution was analyzed using a Dionex ICS-5000 + system (Thermo Fisher Scientific, Sunnyvale, CA, USA) equipped with a pulsed amperometric detector (PAD) and a CarboPac™ PA200 analytical column (3 × 250 mm) coupled to a PA200 guard column (3 × 50 mm) as described by Huang et al. (2023) (Huang et al. 2023). Samples were diluted 1:1000 (v/v) in nanopure water, filtered through a 0.2 μm syringe filter (polyethersulfone, Thermo Scientific™ Nalgene™), and a 25 µL aliquot was injected for analysis. The flow rate was set to 0.5 mL/min.

Mobile phase A was deionized water, and mobile phase B was 200 mM NaOH. Separation was performed under isocratic conditions at 2% B from 0 to 15 min, followed by a linear gradient to 20% B from 15 to 50 min. The column was regenerated at 100% B from 50 to 60 min and re-equilibrated from 60 to 75 min before the subsequent injection. Detection was performed by PAD using a gold working electrode and an Ag/AgCl reference electrode under the standard quadruple waveform recommended by Thermo Fisher Scientific for carbohydrates. Standards in the range Xyl(2)–Xyl(10) were used for retention-time assignment of peaks in the samples. The relative abundance of each oligosaccharide was calculated as a percentage of the total integrated peak area for all detected peaks. Data were collected and processed using Chromeleon™ 7.2 software.

## Definitions of yield and severity factor

XOS yield was calculated based on the amount of XOS produced relative to the initial xylan content of the biomass. The CBA yield was calculated by the amount of CBA produced divided by the amount of cellulose consumed, assuming a molecular weight of 324.3 g/mol for cellulose. Cellulose conversion was calculated from the difference between the initial and residual cellulose divided by the initial cellulose amount.

The hydrothermal severity factor ($\:{R}_{0}$) was calculated by integrating the temperature-dependent severity contribution over the heating period and the 8-min residence period at 180 °C using the following equation.

$$ \begin{gathered} logR_{0} = log_{{10}} \left[ {\int_{{t_{0} }}^{{t_{{hold}} }} {exp\left( {\frac{{T\left( t \right) - 100}}{{14.75}}} \right)dt} } \right. \hfill \\ \left. { + t_{h} \;exp\left( {\frac{{T_{{hold}} - 100}}{{14.75}}} \right)} \right] \hfill \\ \end{gathered}$$

where $\:T\left(t\right)$ is the reactor temperature (°C), $\:{t}_{h}$is the residence time at the holding temperature, $\:{T}_{hold}$ is the holding temperature, and 100 °C and 14.75 °C are the reference temperature and empirical temperature constant, respectively. For each reactor, the temperature during heating was assumed to vary linearly between the specified temperature-time points.

### Statistical analysis

All measurements were performed mostly in triplicate, and the results are presented as mean values with ± standard deviation. A sugar recovery standard was included to account for the potential sugar loss during post-acid hydrolysis. For the response surface analysis, the statistical evaluation was performed using ANOVA (JMP software v18, JMP Statistical Discovery LLC, Cary, NC, USA) to determine p-value, R^2^, lack of fit, and pure error.

## Results and discussion

### Extractive-free biomass

The milled almond shells went through the water and ethanol extraction processes to remove water and ethanol-soluble impurities (Smit et al. 2022). After extraction, 3.6 ± 0.1% of the extractives were removed. The mass compositional analysis of raw and extractive-free almond shells is shown in Table 1. The removal of the extractives proportionally enriched the remaining structural components. The major components were xylan, glucan, lignin, and ash. The remaining fraction, 18.9 ± 0.9 wt% in raw biomass and 19.7 ± 0.9 wt% in extractive-free biomass, may include structural proteins, acetyl groups, pectin, uronic acids, minor sugars, and other minor structural and non-structural soluble components that were not quantified in the present compositional analysis (Sluiter et al. 2011).

**Table 1:** Lignocellulosic content of raw and extractive-free almond shells

Composition (wt%)	Xylan	Glucan	Lignin	Ash	Extractives
Raw biomass	26.3 ± 0.9	26.1 ± 0.1	18.8 ± 0.1	6.3 ± 0.1	3.6 ± 0.1
Extractive-free biomass	27.2 ± 0.9	27.1 ± 0.1	21.6 ± 0.1	4.4 ± 0.3	-

### Optimization of the pretreatment temperature and time

Pretreatment temperature and time were optimized using a response surface central composite design. The pretreatment conditions and the XOS concentration achieved are shown in Table 2. The highest XOS concentration (21.0 ± 2.1 g/L) was obtained at 180 °C for 20 min, comparable to values reported by Morales et al. (2020), who achieved 22.1 g/L XOS at 179 °C (61.4% yield) for 23 min (Morales et al. 2020).

**Table 2:** Experimental design and XOS response for almond shell pretreatment at different temperatures and reaction time levels

Set	Pattern	Temperature (° C)	Time (min)	Xylose (g/L)	XOS (g/L)	Predicted XOS (g/L)
1	+ +	190	30	9.3 ± 0.1	5.9 ± 1.5	6.1
2	− +	170	30	2.4 ± 0.4	19.5 ± 1.3	20.6
3	00	180	20	3.9 ± 0.4	21.0 ± 2.1	20.8
4	00	180	20	3.9 ± 0.3	20.5 ± 0.8	20.8
5	A0	194.1	20	8.5 ± 0.3	10.1 ± 0.7	10.2
6	− −	170	10	0.2 ± 0.0	15.7 ± 1.3	16.5
7	0a	180	5.9	0.6 ± 0.1	20.3 ± 2.6	20.0
8	a0	165.9	20	0.3 ± 0.1	19.8 ± 1.1	18.6
9	+ −	190	10	4.8 ± 0.8	19.2 ± 1.0	19.1
10	0 A	180	34.1	7.3 ± 0.8	14.4 ± 1.3	13.7

The experimental data were fitted to a second-order polynomial model to describe the effects of temperature and reaction time on XOS production. The regression equation describing the relationship between the variables and the XOS concentration is presented in Eq. (2).

2 $$ \begin{gathered} Y_{{XOS}} = \:20.8 - \:3.0\:A - 2.2\:B \hfill \\ - 4.3\:AB\: - 3.2\:A^{2} \: - 2.0\:B^{2} \hfill \\ \end{gathered} $$

Where $\:{Y}_{XOS}$ is XOS concentration; A is temperature, and B is time.

Statistical analysis revealed that all linear, interaction, and quadratic terms significantly affected XOS production (*p* < 0.05) (Table 3). The quadratic regression model was highly significant ( *p* = 0.0014), indicating that the selected process variables effectively explained the variation in XOS production. The model showed an excellent fit to the experimental data, with a coefficient of determination (R²) of 0.98 and an adjusted R² of 0.96. Furthermore, the lack-of-fit was not significant (*p* = 0.2176), indicating that the model adequately described the experimental data and was suitable for predicting XOS production within the investigated experimental range.

**Table 3:** Response surface methodology statistical analysis results

Model term	Parameter Evaluations	Std. Error	*p*-value
Intercept	20.750	0.728	< 0.0001
Temperature (A)	−2.977	0.364	0.0012
Time (B)	−2.230	0.364	0.0036
A × B	−4.275	0.515	0.0011
A²	−3.169	0.482	0.0028
B²	−1.969	0.482	0.0150

The response surface model predicted that the optimal pretreatment conditions for XOS production were 176.7 °C for 17.9 min, with an estimated XOS concentration of 21.5 g/L. However, experimental validation under these predicted conditions yielded an XOS concentration of only 19.8 ± 0.2 g/L and was accompanied by increased xylose formation (1.3 ± 0.1 g/L). Therefore, the pretreatment condition of 180 °C for 20 min was selected instead as the optimal operating condition because it provided more XOS production while maintaining low levels of xylose formation.

### Minimizing xylose formation with time

**Fig. 1:**
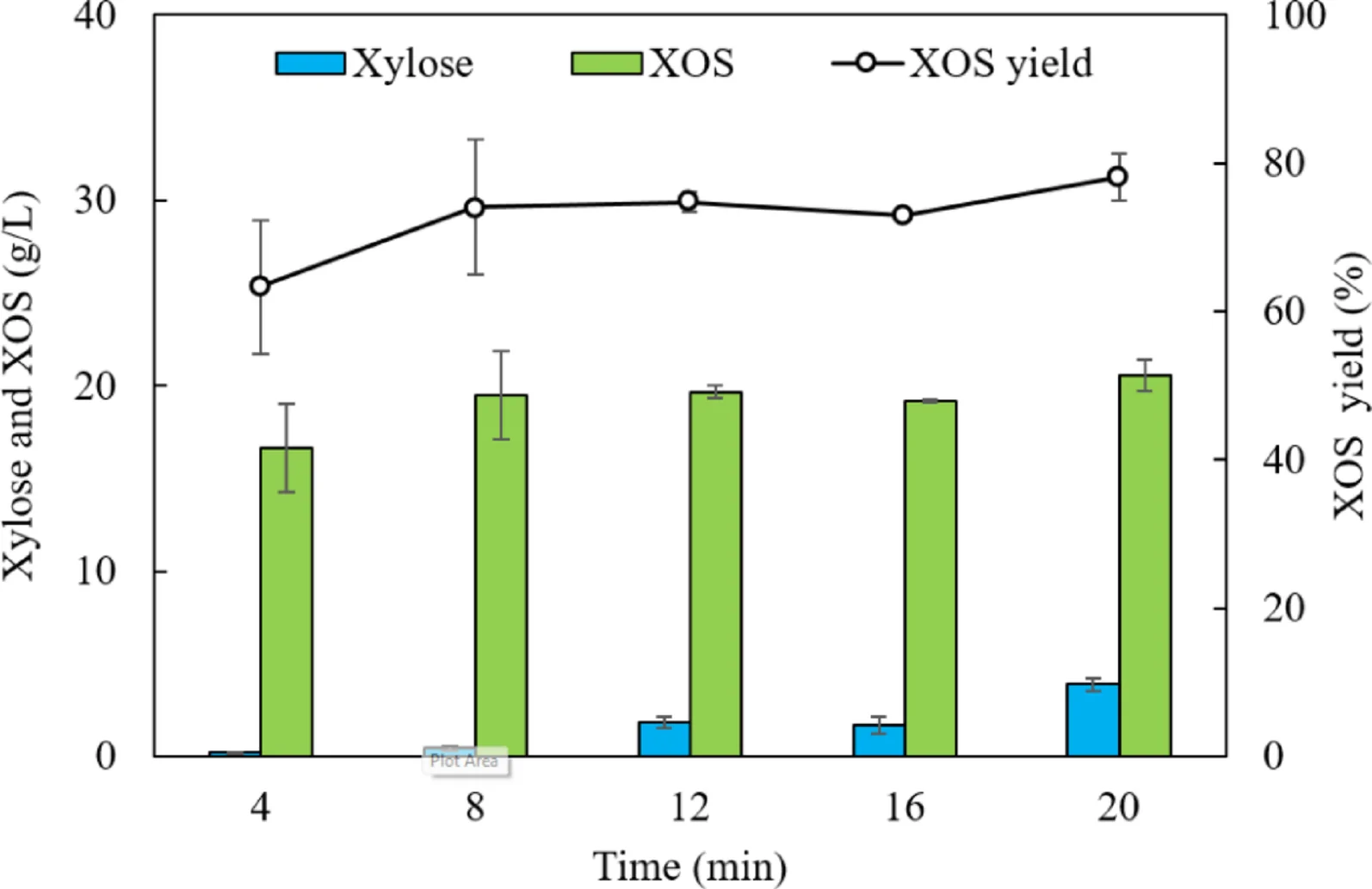
Effect of the residence time on XOS production and yield from almond shell pretreatment; XOS: xylooligosaccharides. All experiments were conducted in triplicate, and error bars represent standard deviation. Pretreatment conditions: solid loading 10% (w/v), temperature 180 °C, and time 4–20 min

Notably, a shorter residence time of 5.9 min at 180 °C produced a comparable XOS concentration (20.3 ± 2.6 g/L) while significantly reducing xylose formation (0.6 ± 0.1 g/L). Because lower xylose production is desirable, residence times ranging from 4 to 20 min at 180 °C were systematically evaluated to examine their effects on XOS and xylose production. As shown in Fig. 1, xylose concentration increased with increasing residence time, whereas XOS concentration remained relatively stable at residence times ≥ 8 min. Although 180 °C for 20 min produced the highest XOS concentration, 180 °C for 8 min was selected as the practical optimum as a compromise between maintaining high XOS production and suppressing the formation of xylose rather than as the absolute mathematical optimum of the RSM model, because it achieved comparable XOS production while substantially minimizing xylose formation.

Following optimization of pretreatment parameters in the tube reactor, the hydrothermal pretreatments were conducted in Parr reactors at 180 °C for 8 min. The 14 mL tube reactor took only 1 min to heat from 100 to 180 °C, whereas the 200 mL and 1 L Parr reactors took 10 and 28 min, respectively, to reach the same temperature. The Parr reactors produced slightly lower XOS concentrations than the tube reactor, yielding 17.4 ± 2.4 g/L and 17.2 ± 1.3 g/L for the 200 mL and 1 L Parr reactors, respectively, compared with 18.1 ± 0.1 g/L for the tube reactor, as shown in Table 4. The lower XOS concentrations, along with the greater formation of xylose and degradation products observed in the Parr reactors, were attributed to their slower heating rates. The slower heating resulted in more severe pretreatment conditions, as indicated by the corresponding severity factors. These results highlight the importance of minimizing thermal lag to improve XOS selectivity and ensure comparable pretreatment conditions across different reactor configurations. Continuous-flow hydrothermal systems or direct steam injections may offer additional opportunities to achieve rapid and uniform heating while improving XOS selectivity (Deng et al. 2025; Makishima et al. 2009).

**Table 4:** Comparative study of the tube reactor and Parr reactor for XOS production from almond shell biomass

Reactor type	Solid loading (%)	Severity factor (log *R*_0_)	Xylose (g/L)	XOS (g/L)	Acetic acid (g/L)	Furfural (g/L)	Formic acid (g/L)	5-HMF (g/L)
Tube reactor	10	3.27	0.4 ± 0.0	18.1 ± 0.1	0.6 ± 0.1	0.1 ± 0.0	0.4 ± 0.0	ND
Parr reactor 200 mL	10	3.35	1.1 ± 0.6	17.4 ± 2.4	1.1 ± 0.1	0.1 ± 0.0	0.4 ± 0.0	ND
Parr reactor 1000 mL	10	3.47	4.9 ± 1.0	17.2 ± 1.3	1.8 ± 0.1	0.4 ± 0.0	0.5 ± 0.0	ND

### Degree of polymerization characterization

**Fig. 2:**
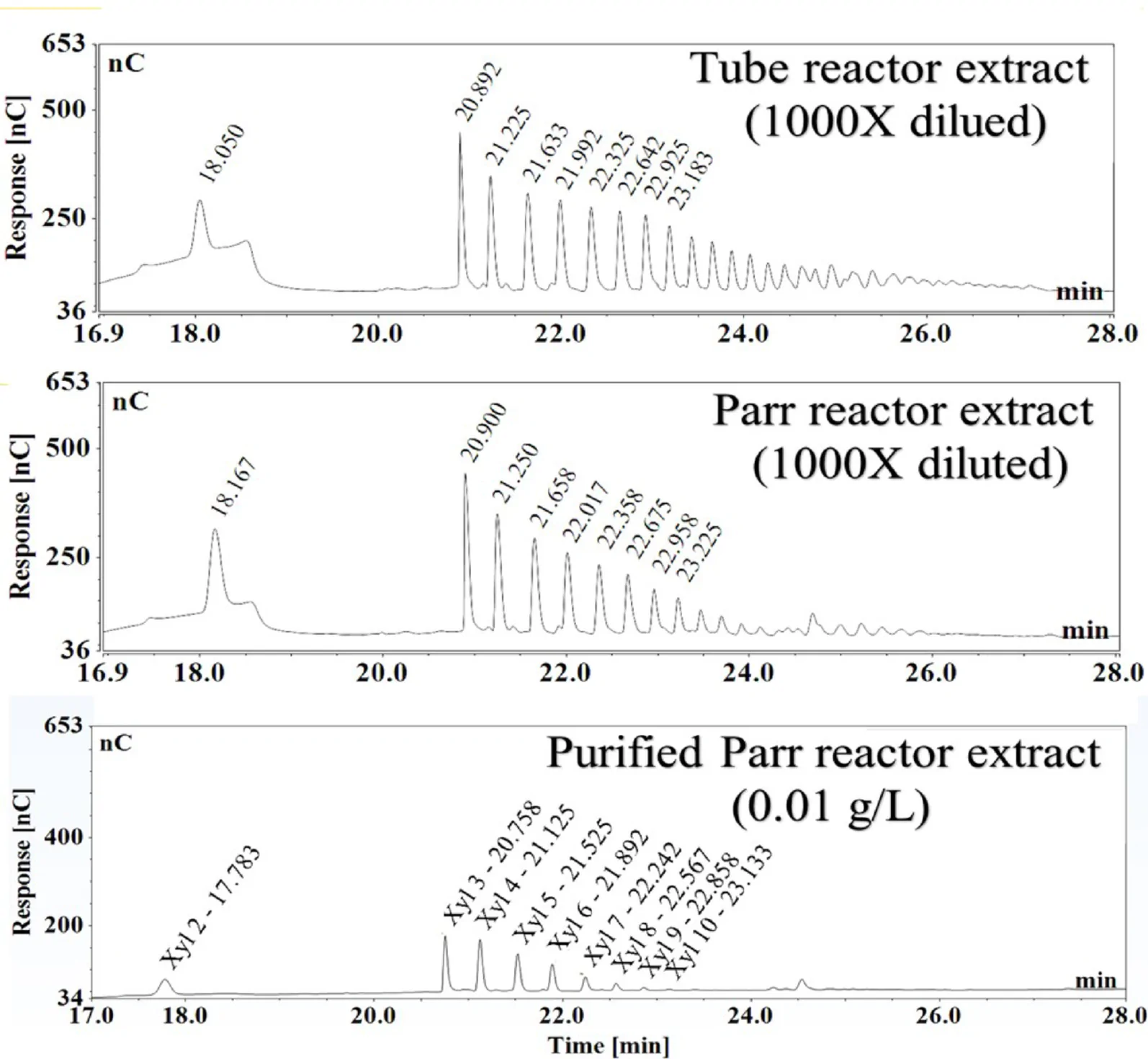

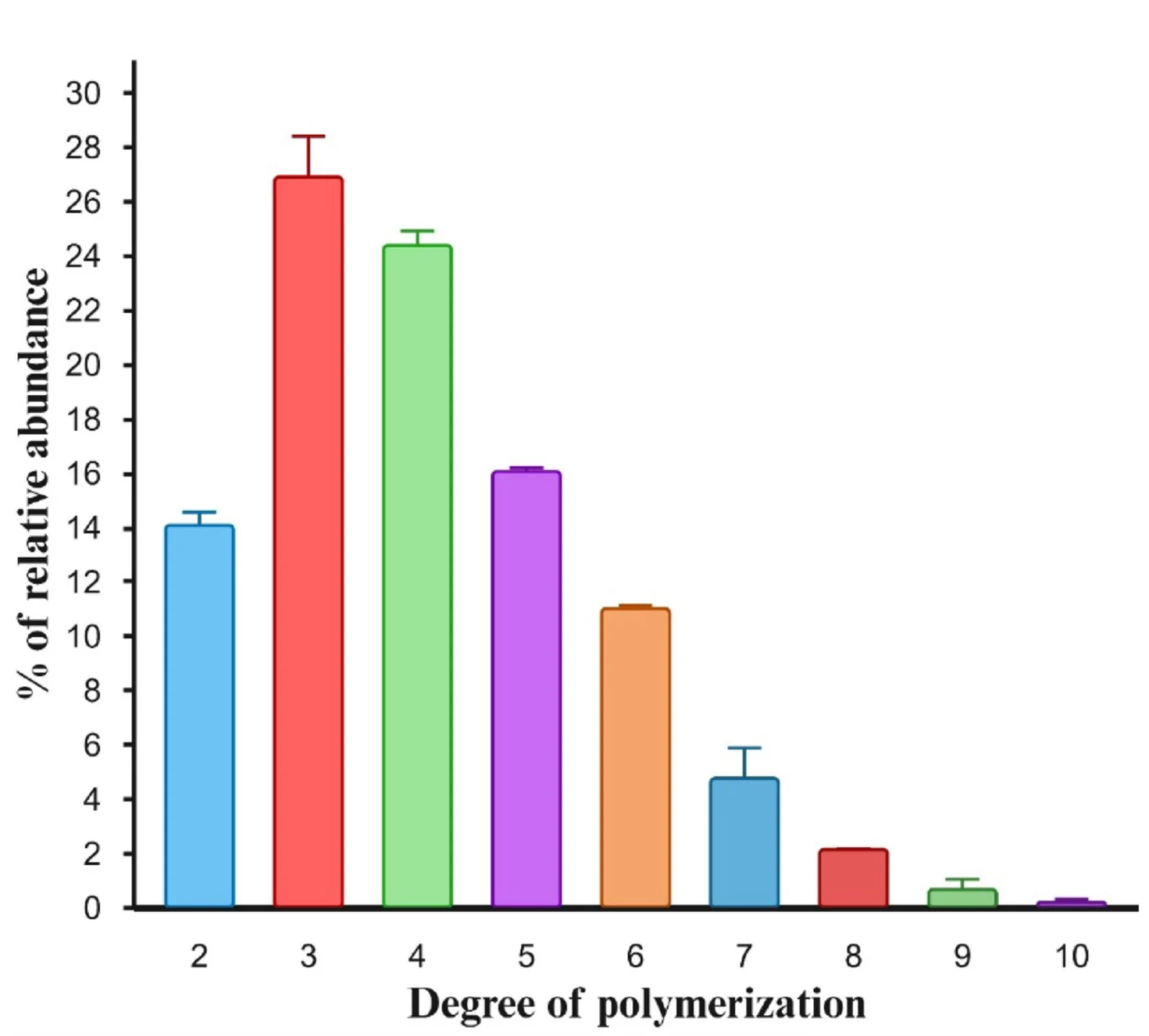
**a** HPAEC–PAD chromatograms of almond shell hydrolysate: (Top) tube-reactor hydrolysate; (middle) Parr-reactor hydrolysate; (bottom) purified Parr-reactor hydrolysate; Labeled peaks correspond to the XOS ladder (Xyl_2_–Xyl_10_); RT ≈ 17–25 min; **b** Relative abundance (%) of xylooligosaccharides (Xyl_2_–Xyl_10_) identified in the purified Parr-reactor hydrolysate

At identical dilution, the tube-reactor hydrolysate showed a denser series of peaks between 21 and 25 min compared with the Parr-reactor hydrolysate (Fig. 2a). This indicates a broader distribution of oligosaccharides, suggesting less controlled hydrolysis and closer elution among oligosaccharides of similar chain lengths. In contrast, the Parr-reactor hydrolysate displayed a more defined XOS ladder, with clear resolution of oligosaccharides from Xyl_2_ to Xyl_10_ (DP2-DP10) and reduced signal intensity at higher DPs. This pattern reflects a more selective depolymerization process, producing a narrower and cleaner distribution of oligosaccharides. After charcoal/ethanol purification, the chromatogram of the Parr-reactor fraction showed distinct, well-separated peaks with minimal background noise. Even at higher injection concentrations, only trace signals were detected beyond DP10, confirming the high purity and compositional uniformity. Quantitative analysis (Fig. 2b) revealed that Xyl_3_ and Xyl_4_ were the predominant oligosaccharides, consistent with the findings reported by other researchers (Sivan et al. 2024; Sonkar et al. 2023), followed by a gradual decrease in the abundance of higher-degree oligosaccharides (Xyl_5_–Xyl_10_). This distribution profile indicates efficient extraction and enrichment of short-chain XOS. These results demonstrate that the Parr reactor pretreatment and subsequent purification effectively yielded a more desirable XOS fraction with a well-defined, functionally relevant DP profile.

### Enzymatic hydrolysis comparison

**Fig. 3:**
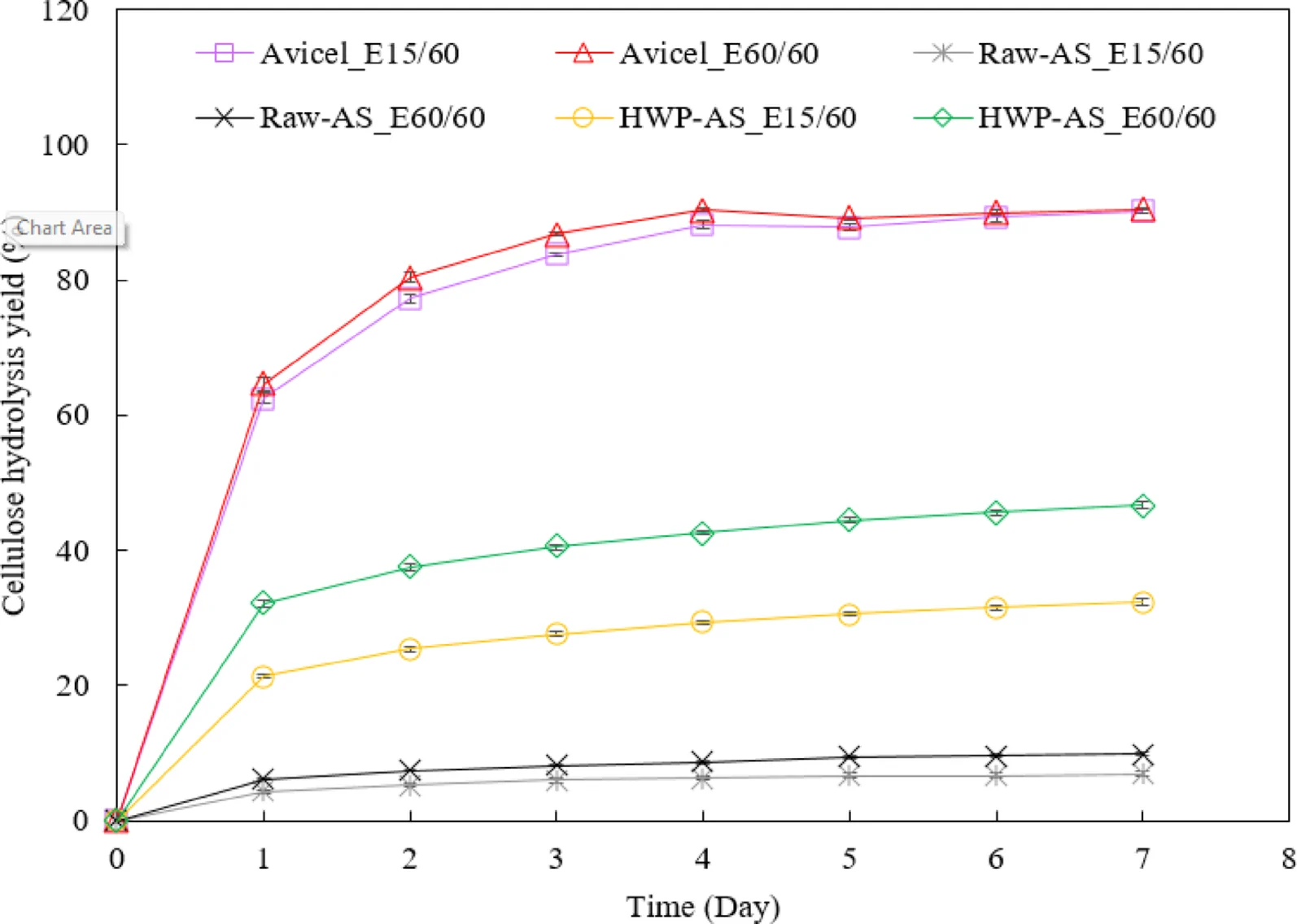
Effect of enzymatic hydrolysis on the cellulose conversion among Avicel, raw almond shells (Raw-AS), and hydrothermal (hot-water) pretreated almond shells (HWP-AS) using a commercial cellulase/β-glucosidase cocktail at two enzyme loadings : (i) E15/60: Enzyme- 15 FPU cellulase, 60 U β-glucosidase/g of cellulose, (ii) E60/60: Enzyme- 60 FPU cellulase, 60 U β-glucosidase/g of cellulose; cellulose-equivalent to 15 g/L; FPU: Filter paper unit. Values are reported as mean ± standard deviation (*n* = 3). Error bars correspond to the standard deviations of triplicate measurements

To assess how pretreatment affects cellulose digestibility, we compared enzymatic hydrolysis of microcrystalline cellulose (Avicel), raw almond shells (Raw-AS), and hydrothermal (hot-water) pretreated almond shells (HWP-AS) using a commercial cellulase/β-glucosidase cocktail at 15 or 60 FPU cellulase (60 U β-glucosidase/g cellulose). Avicel hydrolysis was insensitive to enzyme loading, reaching near-complete conversion (90%) within four days, indicating little recalcitrance to enzymatic hydrolysis. In contrast, Raw-AS showed poor digestibility, yielding only 6.9 ± 0.4% glucose (6.4 ± 0.3 mM) at 15 FPU and 9.8 ± 0.3% (9.1 ± 0.2 mM) at 60 FPU, reflecting strong structural recalcitrance. Pretreatment markedly improved performance: HWP-AS achieved 32.4 ± 0.4% hydrolysis (30.0 ± 0.4 mM) at 15 FPU and 46.6 ± 0.5% (43.2 ± 0.4 mM) at 60 FPU, a 4.75-fold increase over Raw-AS (Fig. 3).

These improvements likely arise from partial hemicellulose removal and disruption of the lignocellulosic matrix, which increase surface area and enzyme accessibility via hydrothermal pretreatment (Basera et al. 2024; Huo et al. 2023; Saratale et al. 2023; Woźniak et al. 2025). However, even at the high enzyme loading, the cellulose conversion stayed as low as 47%, indicating the recalcitrant nature of the pretreated substrate.

### CBA production from the pretreated almond shells

After optimizing pretreatment and hydrolysis conditions, the potential of the recombinant fungus to directly convert hydrothermal-pretreated almond shells into cellobionic acid (CBA) was assessed. This fungal fermentation process integrates enzyme production, cellulose hydrolysis, and CBA biosynthesis within a single system. As a result, it reduces the requirement for externally added enzymes and streamlines biomass conversion.

**Fig. 4:**
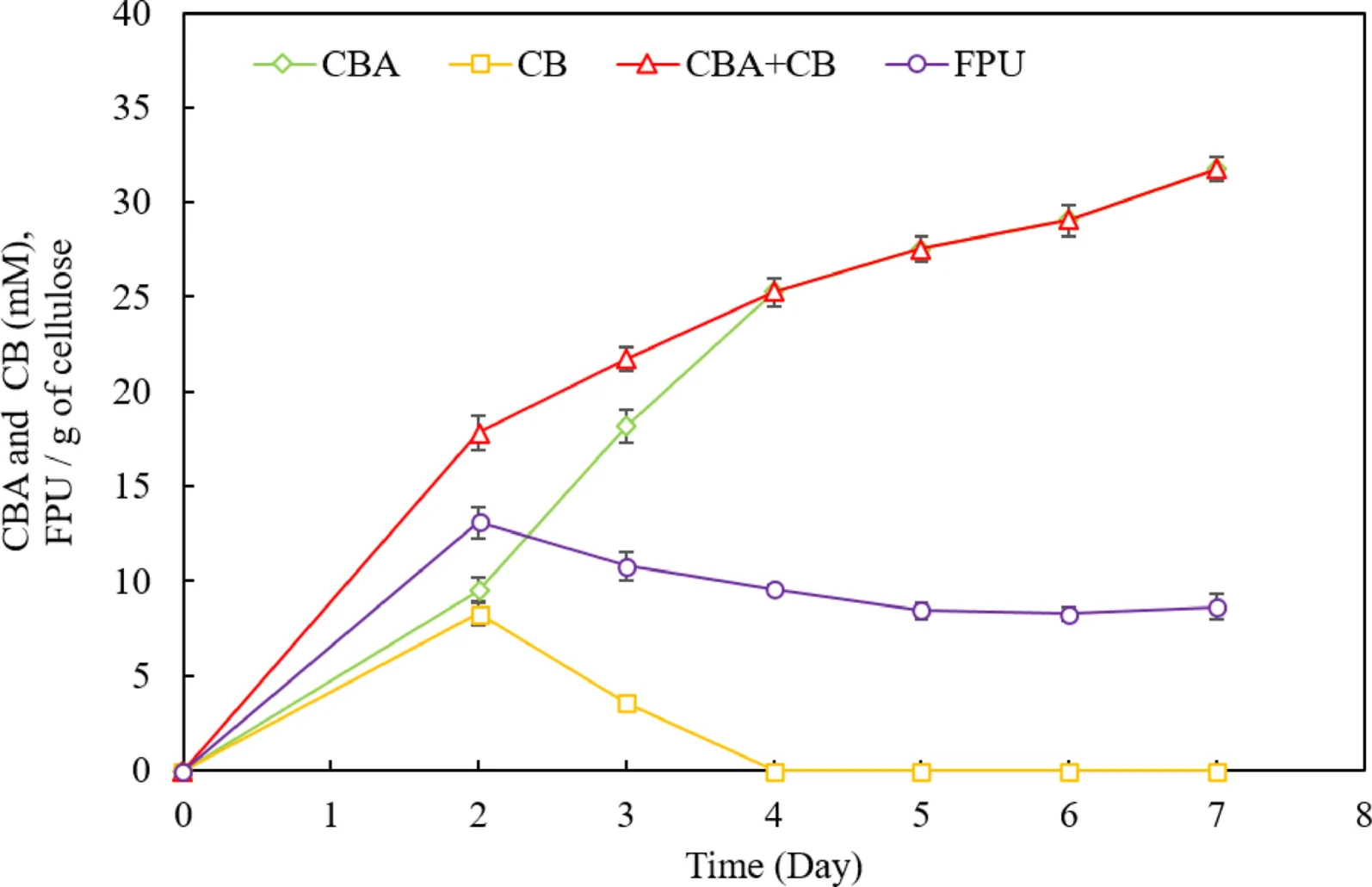
Cellobionic acid (CBA), cellobiose (CB), and cellulase production profiles from hydrothermal (hot-water) pretreated almond shells using *T. heterothallica* TH-11; FPU: Filter paper unit. Experiments were conducted in triplicate (*n* = 3). Error bars represent standard deviations. cellulose-equivalent to 15 g/L, Temp. 40 °C, 230 rpm, 7 days

The hydrothermal pretreated almond shells were used as the substrate for CBA production at a solid loading of 3% (cellulose equivalent 15 g/L). As shown in Fig. 4, the cellobiose titer peaked on day 2 and then gradually decreased to 0, while the CBA titer continued to increase and reached about 31.8 ± 0.6 mM. The cellulose conversion was about 56.4 ± 0.7%, and CBA yield from consumed cellulose was about 89.0 ± 0.5% (Table 5). Maximum cellulase activity (13.1 ± 0.8 FPU/g cellulose) was observed on the 2^nd^ day of fermentation (Fig. 4), indicating that peak hydrolysis occurred within the first three days. During this period, cellulose was efficiently converted into cellobiose and cellobionic acid.

**Table 5:** CBA yield determination from the fermentation of pretreated almond shells

Biomass	Initial cellulose (g)	Residual cellulose (g)	Cellulose conversion (g)	Cellulose conversion (%)	CBA produced (g)	CBA yield from cellulose consumed (%)
Pretreated almond shells	0.74 ± 0.01	0.32 ± 0.01	0.42 ± 0.01	56.36 ± 0.74	0.37 ± 0.01	89.0 ± 0.50

The hydrolysis and cellulose conversion results showed that fungal bioconversion (56.4 ± 0.7%) outperformed commercial enzymatic hydrolysis (60 FPU cellulase, 60 U β-glucosidase/ g of cellulose) of 46.6 ± 0.5% at similar cellulose levels. The superior performance of the fungal system can be attributed to in situ enzyme production, which enables coordinated and adaptive synthesis of cellulases and accessory enzymes in response to the fermentation environment. Moreover, the conversion of cellobiose to CBA alleviated the cellobiose inhibition on the cellulase enzymes (Hildebrand et al. 2016). However, the cellulose conversion and CBA yields were all lower than what was achieved using 2% NaOH-pretreated wheat straw with the recombinant *N. craasa* HL 10 (Wang et al. 2024). This difference can be attributed primarily to the distinct chemical composition and structural characteristics of the two biomass feedstocks, as well as the different mechanisms of hydrothermal and alkaline pretreatment. Their relatively high lignin content and the strong association between lignin, hemicellulose, and cellulose can limit enzyme accessibility to the cellulose fraction (Liu et al. 2016; Yuan et al. 2021). During hydrothermal pretreatment, hemicellulose is preferentially solubilized through the action of heat and autohydrolysis, but lignin is generally not removed as effectively as it is during alkaline treatment. Hydrothermal processing can also cause lignin to redistribute and condense on the biomass surface, forming lignin-rich regions that can hinder cellulase access to cellulose. Although hydrothermal treatment can increase cellulose accessibility by removing part of the hemicellulose fraction, the remaining lignin can continue to act as a physical barrier and can also non-productively adsorb cellulase enzymes. This reduces the effective concentration of enzymes available for cellulose hydrolysis (Saini et al. 2016). The alkaline-treated wheat straw, in comparison, has a more disrupted cell-wall structure and lower lignin-associated recalcitrance, which facilitates enzymatic attack.

To improve cellulose conversion, additional pretreatment strategies can be considered to enhance cellulose accessibility. In addition to chemical approaches for lignin removal, chemical-free physical treatments, such as wet disk refining and intensive milling, offer promising alternatives. These methods can increase the accessible surface area, disrupt the lignin–carbohydrate matrix, and facilitate enzyme penetration, thereby potentially improving enzymatic cellulose conversion (De Assis et al. 2018; Jones et al. 2017).

## Conclusions

This study presents an integrated biorefinery strategy for the valorization of almond shells, an abundant lignocellulosic by-product generated by the agri-food processing industry, into high-value sugar derivatives. Optimized liquid hot-water pretreatment under subcritical conditions enabled efficient hemicellulose depolymerization, producing up to 18.1 g/L of XOS with xylan-to-XOS conversion efficiencies of nearly 69%, while minimizing xylose formation. The resulting hydrolysate was enriched in short-chain XOS (Xyl_3_ and Xyl_4_), spanning a broad distribution from DP2 to DP10. Following pretreatment, the cellulose-rich solid fraction was effectively converted into CBA via fermentation with *T. heterothallica* TH11, achieving > 85% CBA yield at an initial cellulose loading of 15 g/L. Although the pretreatment achieved high xylan-to-XOS conversion, the cellulose conversion to CBA is rather low, indicating that a substantial fraction of the cellulose is not yet fully valorised. Future work will therefore focus on further process optimization, supported by detailed mass and energy balances and techno-economic analysis, to identify key process bottlenecks and improve the overall economic viability of the process. Nevertheless, the demonstrated co-production of XOS and CBA from almond shells provides a promising basis for the further development of an integrated almond-shell biorefinery.

## Supplementary Information

Below is the link to the electronic supplementary material.

Supplementary Material 1

## Data Availability

The data generated during this study can be made available upon reasonable request.

## Abbreviations

ANOVA: Analysis of variance
CBA: Cellobionic acid
CB: Cellobiose
DP: Degree of polymerization
FPU: Filter paper unit
HMF: 5-Hydroxymethylfurfural
HPAEC: PAD-High-performance anion-exchange chromatography with pulsed amperometric detection
ND: Not detected
RSM: Response surface methodology
XOS: Xylooligosaccharides
